# Frontline Healthcare Workers’ Knowledge, Perception and Risk Prevention Practices Regarding COVID-19 in Afghanistan: A Cross-Sectional Study

**DOI:** 10.3390/medsci9010002

**Published:** 2021-01-08

**Authors:** Akshaya Srikanth Bhagavathula, Vijay R. Raghavan, Akbar Ahmadi, Dipankar Srirag, Vijay Kumar Chattu

**Affiliations:** 1 Department of Social and Clinical Pharmacy, Faculty of Pharmacy, Charles University, 50005 Hradec Kralova, Czech Republic; akshaypharmd@gmail.com; 2 The Johanniter International Assistance, Afghanistan Country Programme Kabul, Kabul Dist 10, Afghanistan; Vijay.raghavan@thejohanniter.org (V.R.R.); Akbar.ahmadi@thejohanniter.org (A.A.); 3 Manipal Institute of Technology, Manipal Academy of Higher Education, Manipal 576104, India; dipankarsrirag@icloud.com; 4 Department of Medicine, Faculty of Medicine, University of Toronto, Toronto, ON M5G 2C4, Canada; 5 Department of Public Health Research, Global Institute of Public Health, Thiruvananthapuram 695024, India

**Keywords:** COVID-19, knowledge, perception, practices, healthcare workers, Afghanistan

## Abstract

(1) Background: As of 13 December 2020, Afghanistan reported around 48,952 confirmed COVID-19 cases and 1960 deaths. Lack of knowledge and perceptions among healthcare workers (HCWs) can pose challenges to disease control. Therefore, targeted, timely assessment of knowledge and perceptions are needed to address practices that might hinder efforts to stop the spreading of COVID-19 in Afghanistan. This study aimed to assess COVID-19-related knowledge, perceptions, and risk prevention practices (KPP) among frontline HCWs in Afghanistan; (2) Methods: A cross-sectional study was conducted with the support of field teams who were deployed in Afghanistan, surveyed from 14 to 22 April 2020 in eight provinces in Afghanistan with varying cumulative incidence of COVID-19 cases. A 28-item KPP survey instrument was adapted from other internationally validated questionnaires related to COVID-19. (3) Results: The survey was conducted among 213 frontline HCWs engaged in screening and treating COVID-19 patients. Survey results indicated that basic awareness of COVID-19 was 100% across all the participants. Knowledge and understanding of COVID-19 transmission, symptoms, incubation period and complications associated with COVID-19 are comprehensive and high (>90%), except available treatment for COVID-19 (84%). HCWs’ perceptions towards the prevention and control of COVID-19 were positive. However, only 63% believed that the use of N-95 face masks and disposable and fluid-resistant gowns (76%) could prevent COVID-19 transmission. This survey showed high knowledge and positive perception (72%), and only 48% of frontline HCWs had shown risk prevention practices. Addressing their perceptions and placing additional focus on practices across all health facilities is recommended as a preparedness measure.

## 1. Introduction

The ongoing global COVID-19 pandemic has been characterized as the global health crisis of our time and the most significant challenge we have faced since World War Two [1]. The first confirmed case in Afghanistan was a young man who came from Iran and was admitted to a government hospital in Herat following symptoms of fever, cough, and dyspnea on 22 February 2020 [2]. There have been 48,952 cumulative cases of COVID-19 as of 13 December 2020 [3]. The Capital City (Kabul) and the Western region (Herat province) of Afghanistan are the main areas of interest with high incidences of COVID-19 [4]. 

On 13 March 2020, the Afghanistan COVID-19 Emergency Response and Health Systems Preparedness projects were initiated to strengthen the national system for public health preparedness [5]. The overall project goals are for an emergency response to COVID-19, strengthening the healthcare capacity, mitigating social impact, implementing management and monitoring and evaluation of COVID-19 patients, and a contingent emergency response component (CERC) [5]. The Ministry of Public Health (MoPH), in collaboration with the World Health Organisation (WHO), has developed a COVID 19 Response Plan, and the World Bank approved $400 million to sustain Afghanistan’s reforms momentum to mitigate the COVID 19 crisis. The United Nations Office for the Coordination of Humanitarian Affairs (UNOCHA) launched the COVID-19 Multi-sectoral humanitarian country plan with an estimated $108.1 million appeal. Many international NGOs and United Nations (UN) agencies have responded to COVID 19 in the country [6]. 

The decades of war and political instability in Afghanistan have attenuated the healthcare capacity to detect and control the virus outbreak [7] rapidly. Healthcare workers (HCWs) of all levels are at the frontline of the COVID-19 pandemic response in screening and caring for the infected patients. The occupational risk of exposure to infected individuals and lack of protective measures may pose severe psychological distress; long working hours can lead to fatigue and occupational burnout [8]. A critical aspect of the COVID-19 response was to educate and engage the frontline HCWs in preventing transmission. However, the literature suggests that a poor understanding of the disease can delay the identification and spread of COVID-19 infection [9,10,11]. A study from Italy among the students from life sciences courses showed a higher awareness regarding the infection and the control measures [12]. Another study by Lahner et al. assessed the COVID-19 infection among the health workers in a teaching hospital and found a lower prevalence of COVID-19 than the general population and concluded that high awareness about COVID-19 is mandatory for all, especially for health workers, to safeguard their health and that of patients [13]. Indeed, HCWs’ personal beliefs and experiences can potentially influence patient care during the pandemic period. However, little is known about the personal protection practices such as gloves, surgical face masks, eye protection equipment, and regular hand hygiene activities among HCWs. Therefore, targeted, timely assessment of knowledge and perceptions are needed to address practices that might hinder efforts to stop the spreading of COVID-19 in Afghanistan. Thus, we aimed to assess COVID-19-related knowledge, perceptions, and risk reduction practices (KPP) among HCWs in Afghanistan. 

## 2. Materials and Methods 

**Figure 1:**
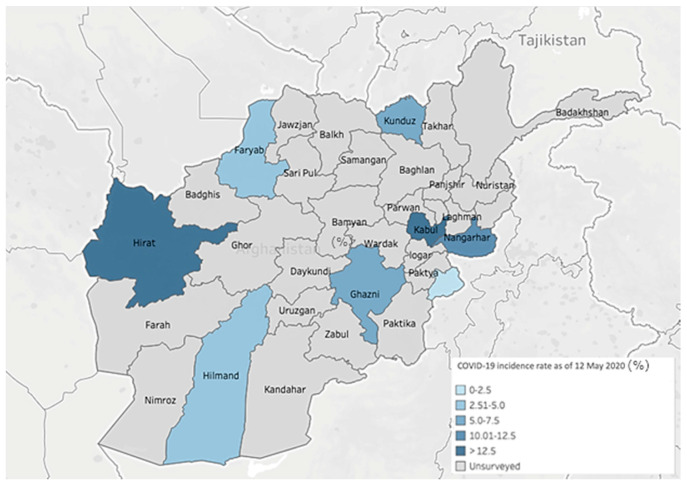
Cumulative incidence of COVID-19 in the surveyed regions-Afghanistan, as of 12 May 2020.

From 14–22 April 2020, a team of trained staff from The Johanniter International Assistance (JIA), in partnership with a locally trained staff of JIA’s partner NGOs, conducted a COVID-19 related KPP cross-sectional survey in eight provinces in Afghanistan (Faryab, Ghazni, Helmand, Herat, Kabul, Khost, Kunduz, and Nangarhar) with varying cumulative incidence of COVID-19 cases per 100,000 population (Figure 1). The selected provinces are stratified based on average incidence rates (12.5 cases) of COVID-19 per 100,000 Afghan population during April 2020. Two of the eight provinces, Herat, and Kabul had high cumulative incidences (≥12.5 cases per 100,000), and the other six provinces had low cumulative incidence rates of COVID-19 (<12.5 cases per 100,000 population). Herat and Kabul reported COVID-19 cases early in the outbreak (March 2020). In early April 2020, JIA and its partners have initiated provisional support to COVID-19-response activities across public health directorates across these eight provinces. 

The present KPP survey is a part of COVID-19 response and preparedness activities in Afghanistan. A snowball sampling design was used in the study by recruiting the frontline HCWs engaged in COVID-19-response activities. Survey areas were selected based on communities with a higher number of newly diagnosed COVID-19 cases and health facilities prepared for treating COVID-19 patients. Across eight provinces, more than 50 survey areas were identified.

### Survey Instrument

The survey questionnaire was adopted from previous studies on the knowledge and perceptions of COVID-19 [9,10,11]. The authors included additional questions about the risk reduction practices of COVID-19 adopted from the WHO interim guidelines on risk assessment and management of COVID-19 [14]. The initial questionnaire was checked for clarity, wordiness, and double-ended questions and was validated for content and relevance by authors and subject experts (public health researchers) using face and content validation methods. The revised questionnaire was assessed for internal reliability, and consistency in a pilot tested on 20 randomly selected HCWs who were not included in the study. The questionnaire had a reliability correlation coefficient of ≥0.76. The internal consistency was assessed by calculating the Cronbach alpha of the knowledge section (0.813), perception section (0.763), and risk reduction practice (0.781) parts in this study. 

The 28-item structured questionnaire consisted of questions that covered several areas: (1) sociodemographic data (3 items: age, gender, occupation); (2) source of information (4 statements/4-point Likert scale: 1-least used to 4-most used); (3) COVID-19 knowledge (7 multiple-choice questions designed to gauge HCWs understanding of COVID-19 symptoms (2 items), transmission (2 items), precautions and risk prevention (3 items)); (4) perceptions of COVID-19 (7 true/false items); (5) risk reduction practices such as the use of personal protective equipment (PPEs) at health facilities and screening sites of COVID-19 patients (7 items; yes/no questions). 

Respondents were considered to have “good” knowledge if they provided “correct” responses to at least four of seven questions related to COVID-19 knowledge and “positive” perception if five out of seven statements are correct. Responses related to the use of risk reduction practices were considered high (≥6 of 7) and low (0–5). 

Subject matter experts reviewed the survey’s content before it was administered and pilot-tested the surveys. Surveyors were instructed to share accurate information about COVID-19 after the survey. The field team approached the HCWs at their workplace, and telephone interviews were conducted among those working in high incidence areas. Surveys were identical for all survey regions and administered in the English language. High-level precautions were taken while conducting the survey when approaching HCWs at their workplace. 

The collected responses were summarized by region, based on COVID-19 incidences (high (Herat and Kabul) versus low (Nangarha, Faryab, Ghazni, Helmand, Khost, and Kunduz)). Categorical variables were presented as absolute numbers and percentages. Continuous variables as mean ± standard deviations (SD) or median (range). To test the differences among the variables, we used the χ2 test, or Fisher’s exact test was used for categorical data. Wilcoxon ran-sum tests were used for ordinal data. Univariate analysis was performed to assess the differences between knowledge, perceptions, and practices among participants’ administrative region; 95% confidence intervals were generated for overall and regional data. Comparisons of survey responses of HCWs between COVID-19 regions described as high-incidence and low-incidence were conducted using the Chi-square (nominal/ordinal data) and Mann–Whitney (continuous variables) tests. We also calculated Cohen’s *d*, a standardized effect size estimation, by taking the differences between two means and dividing them by the pooled standard deviation of the two groups. The differences between the effect estimates were considered small when Cohen’s *d* score (between 0.20 and 0.49), medium (0.5–0.79), and large (≥0.80). 

## 3. Results

Overall, there were 213 HCW respondents from eight provinces (Kabul (*n* = 33), Nangarhar (*n* = 27), 26 each from Faryab, Ghazni, Herat, Khost, and Kunduz, and Helmand (*n* = 23). Although no official records were kept, the average response rate was estimated at >90%, based on the field teams’ experience with the refusal rate of HCWs approached. Among all HCWs, 82 (38%) were women, between 26 and 40 years (51%). Around one-third of them are medical doctors and work in tertiary care hospitals (31%). Everyone (100%) reported that they had heard of COVID-19 before the KPP survey questions about COVID-19 were administered. The summary of respondents’ sociodemographic characteristics is presented in Table 1. 

### 3.1. Knowledge of COVID-19 Cause, Transmission, Signs, and Symptoms

Most of the HCWs from high incidence COVID-19-affected regions (Herat and Kabul) and also the Southern region (Helmand) showed a higher level of knowledge levels than those from the East (Khost and Nangarhar) and Northern region (Faryab and Kunduz) (Table 2). Overall, 67% (95% CI: 60.3–73%, *p* = 0.002) of the HCWs perceived the cause/origin of COVID-19 to be bats. Most HCWs knew that COVID-19 is transmitted by contact, air, and fecal-oral routes (91%, *p* = 0.003). Concerning symptoms, 97% of respondents were aware of headache, fever, cough, sore throat, and flu-like symptoms of COVID-19 infection and knew that COVID-19 symptom onset ranges from 2 to 14 days (97%, *p* < 0.007*), and also reported that COVID-19 could cause pneumonia, respiratory failure, and death. Knowledge about COVID-19 treatment and prevention was high among HCWs such that 84% of respondents expressed that currently, supportive care is the only available treatment for COVID-19.

### 3.2. Source of Receiving COVID-19-Related Information

**Figure 2:**
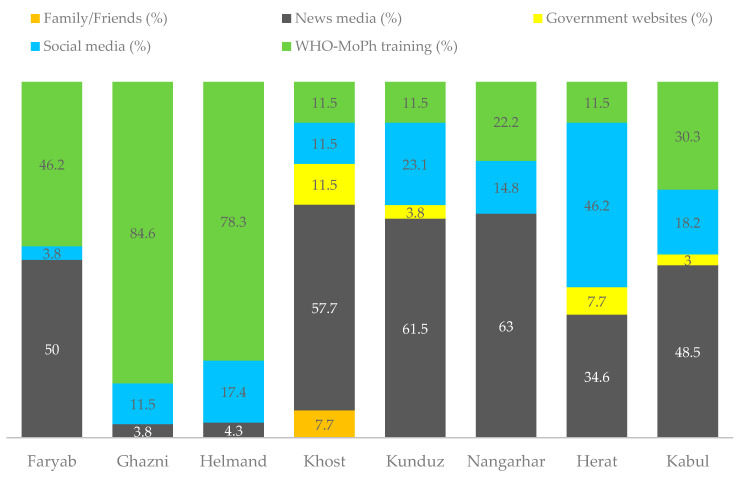
Source of receiving COVID-19-related information.

Training from WHO and MoPH was the primary COVID-19 information channel mentioned by HCWs from Ghazni (84.6%) and Helmand (78.3%) region. The most trusted information source on COVID-19 was the news media in Nangarhar (63%), Kunduz (61.5%), Khost (57.7%), Faryab (50%), and Kabul (48.5%). However, social media is the major source of information in Herat (46.2%) (Figure 2).

### 3.3. Perception of COVID-19 Risk and Prevention

Overall, >90% of HCWs reported that COVID-19 is not fatal (98%), patients should report their travel history first (96%), symptoms appear in 2–14 days (94%), and the flu vaccine is not sufficient for prevention of COVID-19. A vast majority of the respondents also expressed that eating well-cooked meat is safe (89%), and equipment used in the wet market should be disinfected every day (90%). Besides, almost all agreed (99%) that washing hands with soap and water can prevent transmission of COVID-19 (Table 2).

### 3.4. COVID-19-Related Risk Prevention Practices

The majority of participants across all regions (95%) indicated that using gloves (98%), surgical face masks (98%), and regular hand hygiene (95%) practices can prevent the risk of COVID-19 transmission. However, only 63% believed that N-95 face masks and disposable and fluid-resistant gowns (76%) could prevent COVID-19 transmission (Table 2).

### 3.5. KPP in High and Low-Incidence Regions

The knowledge areas where high-incidence provinces scored lower were related to the questions of COVID-19 origin, its transmission, and treatment for COVID-19 patients (Table 3). More HCWs from low-incidence provinces believed that the COVID-19 originated from bats (80% versus 62%), and transmission (100% versus 87%), compared with those from high-incidence provinces. Among respondents from all eight provinces, >90% perceived that COVID-19 symptoms onset in 2–14 days, sick patients should share their recent travel history, and washing hands with soap can prevent COVID-19 transmission. However, respondents from low-incidence regions were not confident in their ability to identify that COVID-19 is not fatal (83%), the flu vaccine is ineffective (83%), and equipment used in the wet market should be disinfected (86%). One statistically significant difference in risk prevention practice between high-and low-incidence regions was use of eye protection glasses (79% (high-incidence) and 93% (low-incidence), *p* = 0.015) (Table 3). 

Overall, HCWs answered a median of 7 (4–7) of 7 COVID-19 knowledge questions correctly, with a slight variation in their knowledge levels between high-incidence regions (median= 7; range: 5–7) versus low-incidence regions (median= 6; range: 4–7). The correct responses for perceptions (median = 6 of 7 questions correctly; range = 4–7), and risk reduction practices (median = 5 of 7 questions correct; range: 0–7) were also moderate, and their scores differed slightly by region. The overall COVID-19-related knowledge was high (98.6%), with moderate perceptions (71.4%) and poor-risk prevention practices (48%) among HCWs, based on cut-off values in each section of the KPP survey. However, a considerable proportion of the frontline HCWs from high-incidence regions reported negative perceptions and poor-risk prevention practices (Table 4). We calculated a Cohen’s *d* of 0.280 (*p* = 0.076) for knowledge scores, 0.012 (*p* = 0.935) for perception scores, and 0.034 (*p* = 0.804) for risk perception practices to prevent COVID-19.

## 4. Discussion

The assessment was the first national-level quantitative evaluation of COVID-19-related knowledge, perceptions, and risk reduction practices among frontline HCWs during a period of ongoing COVID-19 transmission. Overall, COVID-19 knowledge was high, 72% had a positive perception, and only 48% had shown risk prevention practices, based on the correct responses for the 21 statements in the sections of the KPP survey. Simultaneously, this survey revealed several key areas of concern as Afghanistan has recorded a many COVID-19 cases and hundreds of frontline HCWs infected with COVID-19 [15]. Across all regions, HCWs were somewhat less able to correctly recognize the origins of the virus, recommended treatment for COVID-19, use of disposable fluid-resistant gowns, and N-95 face masks to reduce the risk of exposure from COVID-19 infection. The lack of recognition of the origin of COVID-19 might be partially explained by the fact that there is considerable debate on the origin of the causative agent. Given the similarity of SARS-CoV-2 to SARS-CoV, coronaviruses that are ~96% identical, it is likely that bats serve as reservoirs of COVID-19 [16,17]. Yet, questions about the origin of COVID-19 are still unclear. Besides, a quarter (22%) of the frontline HCWs from the low-incidence region and 13% from the high-incidence region believed that COVID-19 patients can be treated with antivirals and that there is a vaccine available. Currently, there is no validated treatment for COVID-19, although some drugs are under investigation and supportive treatment was the only strategy. The targeted educational messages state “that there is no standard treatment for COVID-19, and supportive treatment should be prioritized.” The WHO and MoPH guidelines say that all the frontline HCWs preparing to access suspected or confirmed COVID-19 patients should wear full PPE, including long-sleeved disposable gowns and N95 masks, to protect them against infection [18]. The passiveness risk prevention practices among HCWs in Afghanistan can be due to PPE shortage during the pandemic period. However, the current ongoing COVID-19 epidemic in Afghanistan reinforces the need to practice risk reduction practices among frontline HCWs. 

Although we included various regions with low-and high-incidences of COVID-19, most participants in this geographically diverse sample understood the principal aspects of COVID-19 transmission, prevention, and risk reduction actions to reduce the risk of acquiring COVID-19. This indicates that frontline HCWs may use safe case management and practices to prevent the spreading of COVID-19 in Afghanistan. Regional variations in the epidemic and related response activities might have resulted in the regional differences in knowledge and perceptions, suggesting that targeting health communication at the district level might be more effective than a uniform, national approach. Underlying differences in the cultures, healthcare infrastructure, and education level across different HCWs might have contributed to regional variations in perceptions and behavior, especially regarding risk prevention practices. 

The findings of this study have some limitations. First, the selection of HCWs within the regions was non-random; thus, mostly men and medical doctors participated in the survey. However, healthcare facilities were selected based on the MoPH approved centers for COVID-19 at the time. As a result, the survey covered areas with varying levels of COVID-19 incidence. Second, the survey was conducted during the ongoing epidemic; questions related to risk prevention practices were not validated. Second, a standardized questionnaire was used for the survey, but none of the responses was open-ended. Therefore, limited information was available beyond the binary yes/no or agree/disagree responses. Third, some participants might have provided socially desirable responses aligned to government recommendations rather than actual opinions. Fourth, the sample size was not calculated due to the adoption of a nonprobability sampling technique. Finally, due to physical distancing and some conflicting regions, we conducted our survey as a telephonic or in-person interview with the list of frontline HCWs provided by the MoPH. Thus, we cannot provide more information about the extent of training preparedness and response actions for COVID-19. 

Despite these limitations, the data made it possible to generate preliminary findings that can be shared with several organizations in Afghanistan and the world within a few days of the COVID-19 epidemic. This information can guide the ongoing response and health communication efforts, which can contribute to controlling the epidemic. KPP surveys during an outbreak can provide valuable information for health communication efforts that can contribute to controlling the outbreak at its source, and thereby enhance COVID-19 containment efforts. 

## 5. Conclusions

This survey, conducted at a time when case counts were rapidly increasing in Afghanistan, showed that COVID-19 knowledge among frontline HCWs was high, 72% had a positive perception, and only 48% had shown risk prevention practices. Addressing their perceptions and placing additional focus on practices across all health facilities is recommended as a preparedness measure.

## Figures and Tables

**Table 1:** Sociodemographic characteristics of healthcare workers surveyed on COVID-19 in Afghanistan.

		Region ^†^				
	Total	East	West	Center	North	South
	213	(*n* = 53, 24.8%)	(*n* = 26, 12.2%)	(*n* = 59, 27.7%)	(*n* = 52, 24.4%)	(*n* = 23, 10.8%)
**Province**						
Faryab	26 (12)	-	-	-	26 (12)	-
Ghazni	26 (12)	-	-	26 (12)	-	-
Helmand	23 (11)	-	-	-	-	23 (11)
Herat	26 (12)	-	26 (12)	-	-	-
Kabul	33 (15)	-	-	33 (15)	-	-
Khost	26 (12)	26 (12)	-	-	-	-
Kunduz	26 (12)	-	-	-	26 (12)	-
Nangarhar	27 (13)	27 (13)	-	-	-	-
**Health facility**						
Primary care center	38 (18)	9 (4)	4 (2)	12 (6)	9 (4)	4 (2)
Community health center	44 (21)	9 (4)	4 (2)	6 (3)	12 (6)	13 (6)
Private clinic/hospital	25 (12)	6 (3)	4 (2)	9 (4)	6 (3)	-
District hospital	40 (19)	10 (5)	4 (2)	10 (5)	13 (6)	3 (1)
Tertiary care hospital	66 (31)	19 (9)	10 (5)	22 (10)	12 (6)	3 (1)
**Age (years)**						
18–25	43 (20)	14 (7)	4 (1.5)	12 (6)	12 (6)	1 (0.5)
26–40	109 (51)	26 (12)	17 (8)	33 (15)	24 (11)	9 (4)
40–60	57 (27)	10 (5)	5 (2)	14 (7)	15 (7)	13 (6)
>60	4 (2)	3 (1.5)	-	-	1 (0.5)	-
**Gender**						
Male	131 (62)	30 (14)	13 (6)	37 (17)	29 (14)	22 (10)
Female	82 (38)	23 (11)	1 (5.5)	22 (10)	23 (11)	1 (0.5)
**Occupation**						
Medical Doctor	80 (35)	26 (12)	4 (2)	19 (8)	17 (7)	14 (6)
Nurse	48 (21)	8 (4)	9 (4)	15 (6)	12 (5)	4 (2)
Specialist Doctor	16 (8.5)	2 (1)	1 (0.5)	3 (1)	5 (2)	5 (2)
Midwife	36 (17)	10 (5)	6 (3)	9 (4)	11 (5)	-
Pharmacist	8 (4)	-	1 (0.5)	3 (1)	4 (2)	-
Lab Technician	7 (3.5)	1 (0.5)	2(1)	2 (1)	2 (1)	-
Vaccinator	4 (3)	2 (1)	1 (0.5)	-	1 (0.5)	-
Psychosocial Counsellor	3 (1.5)	2 (1)	1 (0.5)	-	-	-
Physiotherapist	1 (0.5)	-	-	-	1 (0.5)	-
Others	10 (6)	2 (1)	1 (0.5)	1 (0.5)	6 (3)	-

^†^ Rounded off to the nearest integer.

**Table 2:** Knowledge, perception, and practices related to COVID-19 in healthcare workers in Afghanistan (*n* = 213).

Statements	Correct Response		Region					*p*-Value
	Total	% (95% CI)	East (*n*= 53)	West (*n* = 26)	Center (*n* = 59)	North (*n* = 52)	South (*n* = 23)	
**Knowledge**								
COVID-19 is thought to be originated from bats	142	67 (60.3–73)	28 (53)	20 (77)	48 (82)	28 (54)	18 (78)	0.002 **
COVID-19 is transmitted through air, contact, fecal-oral routes	193	91 (86.7–97.4)	42 (79)	26 (100)	58 (98)	45 (98)	22 (96)	0.003 **
Headache, fever, cough, sore throat, and flu are symptoms of COVID-19	206	97 (94.3–99.1)	52 (98)	23 (88)	58 (98)	50 (96)	23 (100)	0.126
The incubation period of COVID-19 is 2–14 days	207	97 (95–99)	47 (89)	26 (100)	59 (100)	52 (100)	23 (100)	0.007 **
COVID-19 leads to pneumonia, respiratory failure, and death	209	98 (96.3–99.9)	52 (98)	25 (96)	59 (100)	52 (100)	23 (100)	0.755
Supportive care is the current treatment for COVID-19	180	84 (79.6–89.4)	47 (89)	22 (85)	47 (80)	43 (83)	21 (91)	0.662
Hand hygiene, covering nose and mouth while coughing, and avoiding sick contact can help in the prevention of COVID-19 transmission	213	100	53 (100)	26 (100)	59 (100)	52 (100)	23 (100)	-
**Perception**								
COVID-19 symptoms appear in 2–14 days	200	94 (90.7–97.1)	47 (89)	24 (92)	58 (98)	48 (92)	23 (100)	0.174
COVID-19 is not fatal	203	95 (92.5–98.1)	51 (96)	23 (88)	57 (97)	49 (94)	23 (100)	0.365
Flu vaccination is not sufficient for preventing COVID-19	195	91 (87.8–95.3)	48 (91)	25 (96)	53 (90)	49 (94)	20 (87)	0.715
During the outbreak, eating well-cooked and safety handled meat is safe	190	89 (85–93.4)	49 (92)	22 (85)	51 (86)	44 (86)	23 (100)	0.438
Sick patients should share their recent travel history with health care professionals	205	96 (93.7–98.8)	50 (94)	25 (96)	58 (98)	49 (96)	23 (100)	0.715
Disinfect equipment and working area in wet markets at least once a day	192	90 (86.1–94.1)	48 (91)	24 (92)	52 (88)	45 (86)	23 (100)	0.450
Washing hands with soap and water can help in the prevention of COVID-19 transmission	212	99 (98.6–100.4)	53 (100)	26 (100)	59 (100)	51 (98)	23 (100)	0.538
**Risk reduction practices**								
Gloves	209	98 (96.3–99.9)	51 (96)	26 (100)	58 (98)	51 (98)	23 (100)	0.570
Surgical Face Masks	208	98 (95.6–99.7)	50 (94.3)	26 (100)	59 (100)	50 (96)	23 (100)	0.227
Disposable and fluid-resistant gowns	161	76 (69.8–81.4)	36 (68)	24 (92)	46 (78)	35 (67)	20 (87)	0.153
Eye Protection	177	80 (74.9–85.6)	36 (68)	24 (92)	54 (91)	42 (81)	21 (91)	0.006 **
Regular hand hygiene	201	94 (91.3–97.5)	51 (96)	26 (100)	56 (95)	48 (92)	20 (87)	0.317
N-95 Face Masks	134	63 (56.4–69.4)	40 (75.5)	18 (69)	33 (56)	25 (48)	18 (78)	0.014 *

Significant at * *p* < 0.05, ** *p* < 0.001.

**Table 3:** Healthcare workers responses to knowledge, perception, and risk prevention practices based on COVID-19 incidences in Afghanistan (*n* = 213).

		COVID-19 Incidence		
Statements	Overall Responses	High Incidence (*n* = 154, 72.3%)	Low Incidence (*n* = 59, 27.6%)	*p*-Value
**Knowledge**				
COVID-19 is thought to be originated from bats	142 (61.4)	95 (62)	47 (80)	0.13
COVID-19 is transmitted through air, contact, fecal-oral routes	193 (83.5)	134 (87)	59 (100)	0.15
Headache, fever, cough, sore throat, and flu are symptoms of COVID-19	206 (89.1)	151 (98)	55 (93)	0.077
The incubation period of COVID-19 is 2–14 days	207 (89.6)	148 (96)	59 (100)	0.085
COVID-19 leads to pneumonia, respiratory failure, and death	209 (90.4)	151 (98)	58 (98)	0.268
Supportive care is the current treatment for COVID-19	180 (77.9)	134 (87)	46 (78)	0.224
Hand hygiene, covering nose and mouth while coughing, and avoiding sick contact can help in the prevention of COVID-19 transmission	213 (100)	154 (100)	59 (100)	-
**Perception**				
COVID-19 symptoms appear in 2–14 days	200 (86.5)	143 (93)	57 (97)	0.306
COVID-19 is not fatal	203 (87.8)	149 (97)	49 (83)	0.106
Flu vaccination is not sufficient for preventing COVID-19	195 84.4)	143 (93)	52 (88)	0.268
During the outbreak, eating well-cooked and safely handled meat is safe	190 (82.2)	137 (89)	53 (90)	0.809
Sick patients should share their recent travel history with health care professionals	205 (88.7)	148 (96)	57 (97)	0.824
Disinfect equipment and working area in wet markets at least once a day	192 (83.1)	141 (92)	51 (86)	0.262
Washing hands with soap and water can help in the prevention of COVID-19 transmission	212 (91.7)	153 (99)	59 (100)	0.535
**Risk Prevention Practice**				
Gloves	209 (90.4)	151 (98)	58 (98)	0.806
Surgical Face Masks	208 (90)	149 (97)	59 (100)	0.161
Disposable and fluid-resistant gowns	161 (69.7)	151 (98)	46 (78)	0.753
Eye Protection	177 (76.6)	122 (79)	55 (93)	0.015
Regular hand hygiene	201 (87)	144 (94)	57 (97)	0.379
N-95 Face Masks	134 (58)	103 (67)	31 (53)	0.052

Significant at *p* < 0.05.

**Table 4:** Summary results of the knowledge, perceptions, and practices survey—Afghanistan, 14–22 April 2020.

Categories/No.	All Respondents		High-Incidence Provinces *		Low-Incidence Provinces ^†^		Effect Size (Cohen’s *d)*	*p*-Value
**Overall scores**	Median scores (IQR)							
Knowledge about COVID-19/7	7	(4–7)	7	(5–7)	6	(4–7)	0.280	0.076
Perceptions/7	6	(4–7)	6	(4–7)	6	(4–7)	0.012	0.935
Risk reduction practices/7	5	(0–7)	5	(0–7)	6	(0–7)	0.034	0.804
**Overall level**	Proportion (%)							
Knowledge (Good = scored ≥ 4)	210	98.6	59	100	151	98.1	0.161	0.280
Perception (Positive = scored ≥ 5)	152	71.4	39	66.1	113	73.4	0.158	0.293
Practice (high = scored ≥ 6)	102	47.9	24	40.7	78	50.6	0.199	0.192

* High incidence provinces = Herat and Kabul. ^†^ Low incidence provinces = Nangarhar, Faryab, Ghazni, Helmand, Khost, and Kunduz. IQR: Interquartile range.

## Data Availability

Data is available on contacting the corresponding author or first authors requesting with valid reason.
